# CircCCND1调节miR-340-5p/TGIF1轴对H446肺癌细胞恶性生物学行为的影响

**DOI:** 10.3779/j.issn.1009-3419.2024.106.05

**Published:** 2024-03-20

**Authors:** Yi DONG, Cuimin ZHU, Xin LIU, Jiwei ZHAO, Qingshan LI

**Affiliations:** 067000 承德，承德医学院附属医院肿瘤科; Department of Oncology, Affiliated Hospital of Chengde Medical College, Chengde 067000, China

**Keywords:** CircCCND1, miR-340-5p/TGIF1, 肺肿瘤, 恶性生物学行为, CircCCND1, miR-340-5p/TGIF1, Lung neoplasms, Malignant biological behavior

## Abstract

**背景与目的:**

肺癌是常见的肺部恶性肿瘤，探究肺癌发生发展的分子机制是当前研究的热点。环状RNA细胞周期蛋白D1（circular RNA Cyclin D1, CircCCND1）在肺癌中高表达，可能是治疗肺癌的潜在靶点。本研究旨在探讨CircCCND1调节miR-340-5p/转化生长因子β诱导因子同源框1（transforming growth factor β-induced factor homeobox 1, TGIF1）轴对肺癌细胞恶性生物学行为的影响。

**方法:**

检测人正常肺上皮细胞BEAS-2B及人肺癌H446细胞中CircCCND1、miR-340-5p及TGIF1 mRNA表达。将体外培养的H446细胞分为对照组、CircCCND1 siRNA组、miR-340-5p mimics组、阴性对照组、CircCCND1 siRNA+miR-340-5p inhibitor组，检测细胞增殖、线粒体膜电位、凋亡、迁移和侵袭情况，以及各组细胞CircCCND1、miR-340-5p、TGIF1 mRNA、BCL2相关X蛋白（BCL2-associated X protein, Bax）、剪切的半胱天冬氨酸蛋白酶3（cleaved Caspase-3）、N-钙黏蛋白（N-cadherin）、E-钙黏蛋白（E-cadherin）、TGIF1蛋白表达，并验证miR-340-5p与CircCCND1、TGIF1的靶向关系。

**结果:**

与BEAS-2B细胞相比，H446细胞CircCCND1、TGIF1 mRNA升高，miR-340-5p表达降低（P<0.05）。敲低CircCCND1或上调miR-340-5p表达可抑制H446细胞增殖、迁移、侵袭，降低TGIF1 mRNA和TGIF1蛋白表达，促进细胞凋亡；下调miR-340-5p可拮抗敲低CircCCND1对H446肺癌细胞恶性生物学行为的抑制作用。CircCCND1可能靶向下调miR-340-5p，miR-340-5p可能靶向下调TGIF1。

**结论:**

敲低CircCCND1可抑制肺癌H446细胞恶性行为，其作用可能是通过调控miR-340-5p/TGIF1轴实现的。

## Abstract (en)

**Background and objective:**

Lung cancer is a common malignant tumor of the lung. To explore the molecular mechanism of the occurrence and development of lung cancer is a hot topic in current research. Cyclic RNA D1 (CircCCND1) is highly expressed in lung cancer and may be a potential target for the treatment of lung cancer. The aim of this study was to investigate the effect of CircCCND1 on the malignant biological behaviors of lung cancer cells by regulating the miR-340-5p/transforming growth factor β-induced factor homeobox 1 (TGIF1) axis.

**Methods:**

The expression of CircCCND1, miR-340-5p, and TGIF1 mRNA in human normal lung epithelial cells BEAS-2B and human lung cancer H446 cells were detected. H446 cells cultured in vitro were randomly divided into control group, CircCCND1 siRNA group, miR-340-5p mimics group, negative control group, and CircCCND1 siRNA+miR-340-5p inhibitor group. Cell proliferation, mitochondrial membrane potential, apoptosis, migration, and invasion were detected, and the expressions of CircCCND1, miR-340-5p, TGIF1 mRNA, BCL2-associated X protein (Bax), cleaved Caspase-3, N-cadherin, E-cadherin, and TGIF1 proteins in each group were detected. The targeting relationship of miR-340-5p with CircCCND1 and TGIF1 was verified.

**Results:**

Compared with BEAS-2B cells, CircCCND1 and TGIF1 mRNA were increased in H446 cells, and miR-340-5p expression was decreased (P<0.05). Knocking down CircCCND1 or up-regulating the expression of miR-340-5p inhibited the proliferation, migration and invasion of H446 cells, decreased the expression of TGIF1 mRNA and TGIF1 protein, and promoted cell apoptosis. Down-regulation of miR-340-5p could antagonize the inhibitory effect of CircCCND1 knockdown on the malignant biological behavior of H446 lung cancer cells. CircCCND1 may target the down-regulation of miR-340-5p, and miR-340-5p may target the down-regulation of TGIF1.

**Conclusion:**

Knocking down CircCCND1 can inhibit the malignant behaviors of lung cancer H446 cells, which may be achieved through the regulation of miR-340-5p/TGIF1 axis.

肺癌是最常见的肺部恶性肿瘤，已成为威胁人类生命健康的重大卫生问题^[1⇓-3]^。环状RNA细胞周期蛋白D1（circular RNA Cyclin D1, CircCCND1）是一种源自CCND1的新型环状RNA分子，也是癌症发生发展的关键调控因子之一，在喉鳞状细胞癌中上调，并与其侵袭性和不良预后密切相关，CircCCND1的缺失可在体内外抑制喉鳞状细胞癌细胞的生长^[4]^，敲低CircCCND1可减弱非小细胞肺癌的化学耐药性，进而抑制顺铂处理下非小细胞肺癌细胞的增殖并诱导其凋亡^[5]^。miR-340-5p是一种具有抗癌作用的微小RNA分子，可被环状RNA调控，过表达miR-340-5p可抑制透明细胞癌细胞的生长及进展^[6]^，并可有效抑制结直肠癌细胞的增殖、迁移和侵袭以及体内生长^[7]^。Li等^[8]^研究表明miR-340-5p的上调抑制了肺癌细胞的增殖、迁移和血管生成。转化生长因子β诱导因子同源框1（transforming growth factor β-induced factor homeobox 1, TGIF1）作为一种癌基因可促进恶性肿瘤的病理过程，TGIF1的高表达是神经胶质瘤患者预后不良的重要预测因素，敲低TGIF1可抑制神经胶质瘤细胞的增殖和侵袭^[9]^；TGIF1的沉默促进了食管癌细胞凋亡并抑制了其迁移、侵袭和上皮间充质转化（epithelial mesenchymal transition, EMT）过程^[10]^；增强TGIF1基因表达可促进肺腺癌H157细胞的迁移、侵袭和转移，此过程可通过沉默TGIF1来进行阻断^[11]^。生物信息学预测发现miR-340-5p序列与CircCCND1、TGIF1序列存在结合位点，因此预测CircCCND1可能通过调节miR-340-5p/TGIF1轴介导肺癌细胞的恶性生物学行为，本文旨在通过体外人小细胞肺癌H446细胞来验证此预测。

## 1 材料与方法

### 1.1 实验材料与仪器

人正常肺上皮细胞BEAS-2B、人小细胞肺癌H446细胞，购自武汉尚恩生物技术有限公司；一步法逆转录-实时荧光定量聚合酶链式反应（polymerase chain reaction, PCR）试剂盒，购自河北三狮生物科技有限公司；TRIzol RNA提取试剂、兔源抗人Anti-Ki67、Anti-TGIF1、Anti-Bax、Anti-Cleaved Caspase-3、Anti-β-actin、Anti-N-cadherin及Anti-E-cadherin一抗、JC-1检测试剂盒，购自美国Thermo Fisher Scientific公司；Alexa Fluor^TM^ 488标记小鼠抗兔二抗、HRP标记小鼠抗兔二抗、TUNEL检测试剂盒，购自美国Abcam公司；Q3200实时荧光定量PCR仪，购自杭州柏恒科技有限公司；Delphi-X倒置荧光显微镜，购自美国AMSCOPE公司；DxFLEX流式细胞仪，购自美国Beckman公司；HT-Mini01/Mini04/ZY02蛋白电泳与转印系统、HT-600C/600D通用型电泳仪电源，购自江苏天通设备科技有限公司；PRESA-96全自动酶标仪，购自普睿博仪器（杭州）有限公司等。

### 1.2 方法

#### 1.2.1 实时荧光定量PCR实验检测BEAS-2B及H446细胞中CircCCND1、miR-340-5p、TGIF1 mRNA表达

在39.8 ^o^C温水浴中迅速解冻购买的BEAS-2B及H446细胞，分别以DMEM和RPMI-1640培养基进行复苏培养（两种培养基内均混有10%胎牛血清和1%青链霉素双抗）。收集细胞提取总RNA后，按照一步法逆转录-实时荧光定量PCR试剂盒说明书中方法进行PCR，采用2^-ΔΔCt^算法分析各基因循环阈值，并采用GAPDH作CircCCND1、TGIF1的内参对照，同时采用U6作miR-340-5p的内参对照。引物序列见表1。

**表1:** 引物序列

Genes	Direction	Sequence (5'-3')
CircCCND1	F R	TCCTCTCCAAAATGCCAGAG ACTCTGCTGCTCGCTGCTAC
TGIF1	F R	GGATTGGCTGTATGAGCACCGT GCCATCCTTTCTCAGCATGTCAG
GAPDH	F R	AATGGGCAGCCGTTAGGAAA TGAAGGGGTCATTGATGGCA
miR-340-5p	F R	GCGGTTATAAAGCAATGAGA GTGCGTGTCGTGGAGTCG
U6	F R	GCTTCGGCAGCACATATACTAAAAT CGCTTCACGAATTTGCGTGTCAT

CircCCND1: circular RNA Cyclin D1; TGIF1: transforming growth factor β-induced factor homeobox 1; miR-340-5p: microRNA-340-5p.

#### 1.2.2 分组转染H446细胞

将H446细胞接种于24孔板进行传代培养，直至进入对数生长期后随机分为对照组、CircCCND1 siRNA组、miR-340-5p mimics组、阴性对照组、CircCCND1 siRNA+miR-340-5p inhibitor组，采用脂质体3000进行转染，转染24 h后行后续实验检测。

#### 1.2.3 实时荧光定量PCR实验检测各组H446细胞CircCCND1、miR-340-5p及TGIF1 mRNA表达

收集1.2.2中分组转染后的各组H446细胞，使用TRIzol RNA提取试剂提取其总RNA，然后进行实时荧光定量PCR实验来检测各组细胞CircCCND1、miR-340-5p及TGIF1 mRNA相对表达，按1.2.1中所述方法进行操作。

#### 1.2.4 Ki67免疫荧光染色检测各组H446细胞增殖率

采用PBS缓冲液漂洗1.2.2中转染后的各组H446细胞，然后加入10%甲醛溶液覆没细胞，于室温下固定30 min后依次以5%牛血清白蛋白封闭、稀释100倍的兔抗人Anti-Ki67一抗孵育、PBS缓冲液漂洗、稀释150倍的小鼠抗兔二抗（Alexa Fluor^TM^ 488标记）避光孵育，荧光显微镜下观察并拍照，计数Ki67阳性细胞数与细胞总数，计算细胞增殖率（Ki67阳性细胞数/细胞总数×100%）。

#### 1.2.5 检测各组U20S细胞线粒体膜电位

采用PBS缓冲液漂洗1.2.2中转染后的各组H446细胞，计数测定其密度后每组各取含1×10^7^个细胞的细胞悬液于室温下进行离心（300 g, 5 min），所得细胞沉淀采用 JC-1工作液进行重悬孵育，在同样条件下再次离心后用适量PBS缓冲液洗涤、悬浮，混匀后用流式细胞仪检测，并用FlowJo V10软件处理获得各组细胞红色和绿色荧光强度后算出各组细胞线粒体膜电位，公式：细胞线粒体膜电位=红色荧光强度/绿色荧光强度。

#### 1.2.6 TUNEL染色实验检测各组H446细胞凋亡率

采用PBS缓冲液漂洗1.2.2中转染后的各组H446细胞，然后加入10%甲醛溶液覆没细胞，于室温下固定30 min后以TUNEL工作液孵育、PBS缓冲液漂洗，最后在荧光显微镜下观察并拍照，计数各组TUNEL阳性细胞数与细胞总数，计算细胞凋亡率（TUNEL阳性细胞数/细胞总数×100%）。

#### 1.2.7 划痕实验、Transwell侵袭实验分别检测各组H446细胞迁移比、侵袭数

划痕实验：采用PBS缓冲液漂洗1.2.2中转染后的各组H446细胞，计数测定其密度后每组各取含1×10^7^个细胞的细胞悬液于室温下进行离心（300 g, 5 min），所得细胞以无胎牛血清的RPMI-1640培养基悬浮后于24孔板内培养，待细胞铺满孔底后，用无菌枪头在孔底划线，并用Image J软件定量图像中各组划痕面积并以S1表示，加入无胎牛血清的RPMI-1640培养基继续培养24 h后再次采集各组细胞图像，测出其划痕面积并以S2表示，然后算出各组细胞迁移比，细胞迁移比=（S1-S2）/S1×100%。Transwell侵袭实验：采用PBS缓冲液漂洗1.2.2中转染后的各组H446细胞，计数测定其密度后每组各取含1×10^7^个细胞的细胞悬液于室温下进行离心（300 g, 5 min），所得细胞以无胎牛血清的RPMI-1640培养基悬浮（5×10^5^个细胞/mL）后，在Transwell上室内添加100 μL细胞悬液，600 μL培养基（含血清）加入下室，培养24 h后，将细胞固定，进行吉姆萨染色后，显微镜下观察并拍照，用Image J软件定量图像中各组下室内总细胞数，可获得各组细胞侵袭数。

#### 1.2.8 免疫印记实验检测各组H446细胞凋亡与EMT相关蛋白表达、TGIF1蛋白表达

收集1.2.2中转染后的各组H446细胞分别与RAPI裂解液混匀，按照其说明书中方法提取各组总蛋白后通过BCA法测出其浓度，根据结果每组取30 µg总蛋白样品于蛋白电泳与转印系统内进行电泳后湿转，所得膜上蛋白浸没在3%牛血清白蛋白内封闭后依次以均稀释2000倍的兔源抗人Anti-TGIF1、Anti-Bax、Anti-cleaved Caspase-3、Anti-β-actin、Anti-N-cadherin及Anti-E-cadherin一抗孵育、TBST缓冲液漂洗、以稀释1000倍的二抗孵育后显影，Image J软件分析灰度值，计算蛋白相对表达，公式：蛋白相对表达=检测蛋白（TGIF1、Bax、cleaved Caspase-3、N-cadherin、E-cadherin）/内参蛋白（β-actin）。

#### 1.2.9 双荧光素酶报告实验验证H446细胞CircCCND1对miR-340-5p、miR-340-5p对TGIF1的靶向关系

于24孔板内培养H446细胞，将CircCCND1、TGIF1野生型（WT-CircCCND1、WT-TGIF1）和突变型（MUT-CircCCND1、MUT-TGIF1）质粒分别与miR-NC（或miR-340-5p mimics）进行共转染，采用脂质体3000并按照其说明书中方法转染24 h后，测定荧光素酶活性。

### 1.3 统计分析

统计分析采用GraphPad Prism 8.0软件，本实验数据使用均数±标准差（Mean±SD）进行表示，两组间比较采用t检验，多组间比较采用单因素方差分析，多组两两比较采用SNK-q检验。P<0.05为差异有统计学意义。

## 2 结果

### 2.1 人小细胞肺癌H446细胞CircCCND1、miR-340-5p及TGIF1 mRNA表达

H446细胞中CircCCND1、TGIF1 mRNA表达高于BEAS-2B细胞，miR-340-5p表达低于BEAS-2B细胞（P<0.05）。见表2。

**表2:** 各细胞CircCCND1及miR-340-5p表达、TGIF1 mRNA相对表达水平（Mean±SD, n=6）

Groups	CircCCND1	miR-340-5p	TGIF1 mRNA
BEAS-2B	0.98±0.16	1.03±0.17	1.00±0.21
H446	2.17±0.11^a^	0.25±0.08^a^	2.04±0.19^a^

^a^P<0.05 vs BEAS-2B cells.

### 2.2 各组H446细胞CircCCND1、miR-340-5p及TGIF1 mRNA表达

CircCCND1 siRNA组CircCCND1、TGIF1 mRNA表达低于对照组，miR-340-5p表达高于对照组（P<0.05）；miR-340-5p mimics组TGIF1 mRNA表达低于对照组，miR-340-5p表达高于对照组（P<0.05）；CircCCND1 siRNA+miR-340-5p inhibitor组miR-340-5p表达低于CircCCND1 siRNA组，TGIF1 mRNA表达高于CircCCND1 siRNA组（P<0.05）。见表3。

**表3:** 各组细胞CircCCND1、miR-340-5p及TGIF1 mRNA表达（Mean±SD, n=6）

Groups	CircCCND1	miR-340-5p	TGIF1 mRNA
Control	1.00±0.15	1.03±0.16	1.01±0.17
CircCCND1 siRNA	0.31±0.10^a^	2.21±0.20^a^	0.36±0.11^a^
miR-340-5p mimics	0.95±0.14	2.28±0.21^a^	0.32±0.10^a^
Negative control	1.02±0.16	1.01±0.17	1.04±0.15
CircCCND1 siRNA+miR-340-5p inhibitor	0.33±0.11	1.08±0.19^b^	0.96±0.13^b^

^a^P<0.05 vs Control group; ^b^P<0.05 vs CircCCND1 siRNA group.

### 2.3 各组H446细胞增殖、线粒体膜电位与凋亡

CircCCND1 siRNA组、miR-340-5p mimics组细胞增殖率、线粒体膜电位低于对照组，凋亡率高于对照组（P<0.05）；CircCCND1 siRNA+miR-340-5p inhibitor组细胞增殖率、线粒体膜电位高于CircCCND1 siRNA组，凋亡率低于CircCCND1 siRNA组（P<0.05）。见图1-3和表4。

**图1:**
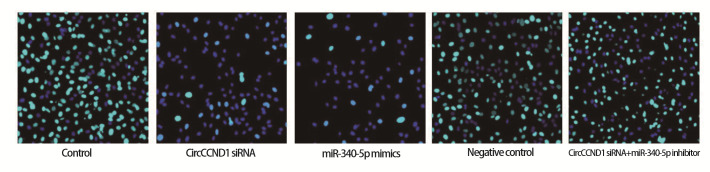
各组H446细胞增殖情况（Ki67免疫荧光染色，×200）

**图2:**
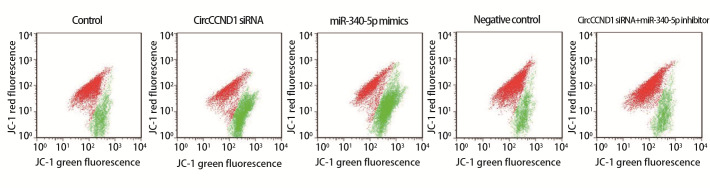
流式细胞实验检测各组H446细胞线粒体膜电位（×200）

**图3:**
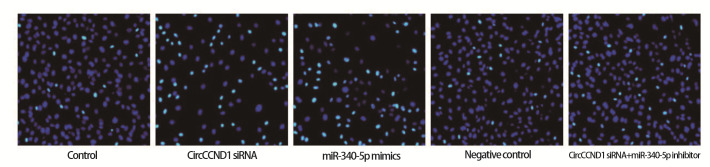
TUNEL染色检测各组H446细胞凋亡率（×200）

**表4:** 各组H446细胞增殖率、线粒体膜电位、凋亡率（Mean±SD, n=6）

Groups	Proliferation rate（%）	Mitochondrial membrane potential	Apoptosis rate（%）
Control	78.25±10.85	1.61±0.22	7.46±2.32
CircCCND1 siRNA	21.90±7.21^a^	0.58±0.17^a^	46.15±3.86^a^
miR-340-5p mimics	18.45±6.10^a^	0.53±0.16^a^	50.24±3.75^a^
Negative control	75.90±9.55	1.64±0.21	7.05±2.33
CircCCND1 siRNA+miR-340-5p inhibitor	70.14±10.35^b^	1.54±0.18^b^	8.96±2.50^b^

^a^P<0.05 vs Control group; ^b^P<0.05 vs CircCCND1 siRNA group.

### 2.4 各组H446细胞迁移、侵袭 CircCCND1

siRNA组、miR-340-5p mimics组细胞迁移比、侵袭数低于对照组（P<0.05）；CircCCND1 siRNA+miR-340-5p inhibitor组上述指标高于CircCCND1 siRNA组（P<0.05）。见图4、5和表5。

**图4:**
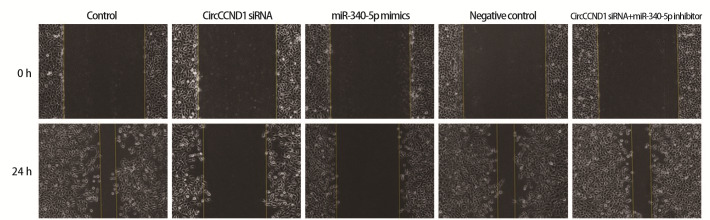
细胞划痕实验检测H446细胞迁移（×200）

**图5:**
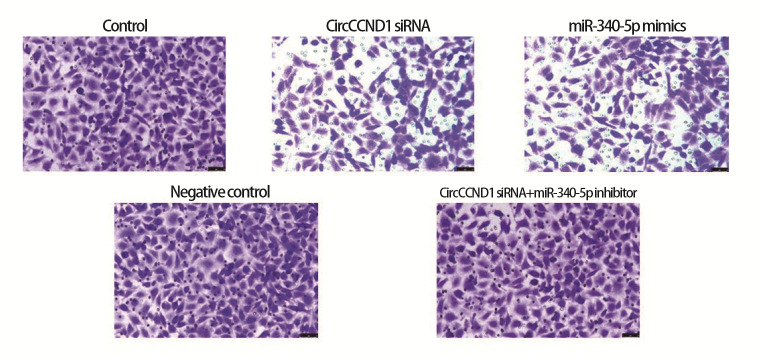
Transwell实验检测H446细胞侵袭（结晶紫染色，×200）

**表5:** 各组H446细胞迁移比、侵袭数（Mean±SD, n=6）

Groups	Migration ratio (%)	Invasion number
Control	83.72±10.85	286.53±30.45
CircCCND1 siRNA	24.97±7.29^a^	90.14±26.21^a^
miR-340-5p mimics	21.04±6.16^a^	82.30±25.85^a^
Negative control	84.18±9.93	298.57±29.70
CircCCND1 siRNA+miR-340-5p inhibitor	80.01±9.42^b^	274.20±28.35^b^

^a^P<0.05 vs Control group; ^b^P<0.05 vs CircCCND1 siRNA group.

### 2.5 各组H446细胞凋亡与EMT相关蛋白、TGIF1蛋白表达

CircCCND1 siRNA组、miR-340-5p mimics组N-cadherin、TGIF1蛋白水平低于对照组，Bax、Cleaved Caspase-3、E-cadherin蛋白水平高于对照组（P<0.05）；CircCCND1 siRNA+miR-340-5p inhibitor组N-cadherin、TGIF1蛋白水平高于CircCCND1 siRNA组，Bax、Cleaved Caspase-3、E-cadherin蛋白水平低于CircCCND1 siRNA组（P<0.05）。见图6和表6。

**图6:**
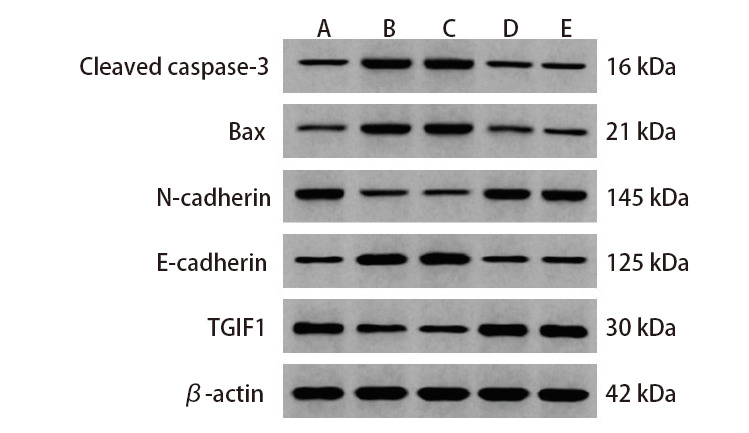
免疫印迹检测各组细胞凋亡与EMT相关蛋白、TGIF1蛋白表达。A：对照组；B：CircCCND1 siRNA组；C：miR-340-5p mimics组；D：阴性对照组；E：CircCCND1 siRNA+miR-340-5p inhibitor组。

**表6:** 各组H446细胞凋亡与EMT相关蛋白、TGIF1蛋白相对表达（Mean±SD, n=6）

Groups	Cleaved caspase-3	Bax	N-cadherin	E-cadherin	TGIF1
Control	0.27±0.08	0.18±0.05	0.69±0.07	0.22±0.07	0.85±0.15
CircCCND1 siRNA	0.72±0.13^a^	0.69±0.10^a^	0.17±0.05^a^	0.73±0.10^a^	0.32±0.10^a^
miR-340-5p mimics	0.74±0.12^a^	0.71±0.11^a^	0.14±0.04^a^	0.76±0.12^a^	0.29±0.09^a^
Negative control	0.25±0.08	0.17±0.05	0.70±0.08	0.21±0.06	0.87±0.14
CircCCND1 siRNA+miR-340-5p inhibitor	0.29±0.09^b^	0.20±0.06^b^	0.65±0.06^b^	0.25±0.08^b^	0.81±0.12^b^

^a^P<0.05 vs Control group; ^b^P<0.05 vs CircCCND1 siRNA group.

### 2.6 CircCCND1对H446细胞miR-340-5p、miR-340-5p对H446细胞TGIF1的靶向调节

查询Starbase数据库可发现CircCCND1与miR-340-5p存在互补位点（图7）。miR-340-5p mimics+WT-CircCCND1组荧光素酶活性低于miR-NC+WT-CircCCND1组（P<0.05），见表7。

**图7:**
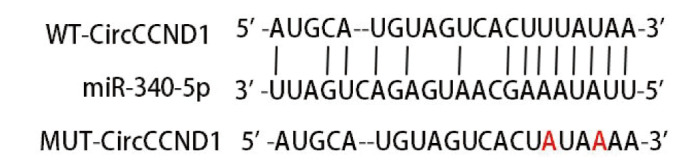
查询Starbase数据库得到的CircCCND1与miR-340-5p之间结合位点（红色表示突变位点）

**表7:** 各组相对荧光素酶活性（Mean±SD, n=6）

Groups	Relative luciferase activity
miR-NC+WT-CircCCND1	1.01±0.13
miR-340-5p mimics+WT-CircCCND1	0.30±0.08^a^
miR-NC+MUT-CircCCND1	0.99±0.12
miR-340-5p mimics+MUT-CircCCND1	0.97±0.11

^a^P<0.05 vs miR-NC+WT-CircCCND1 group.

查询Starbase数据库可发现miR-340-5p与TGIF1之间存在结合位点（图8）。WT-TGIF1+miR-340-5p mimics组荧光素酶活性低于WT-TGIF1+miR-NC组（P<0.05），见表8。

**图8:**
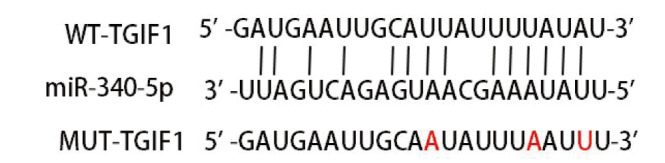
查询Starbase数据库得到得到的miR-340-5p与TGIF1之间结合位点（红色表示突变位点）

**表8:** 各组相对荧光素酶活性（Mean±SD, n=6）

Groups	Relative luciferase activity
WT-TGIF1+miR-NC	0.96±0.12
WT-TGIF1+miR-340-5p mimics	0.24±0.07^a^
MUT-TGIF1+miR-NC	0.97±0.10
MUT-TGIF1+miR-340-5p mimics	1.00±0.11

^a^P<0.05 vs WT-TGIF1+miR-NC group.

## 3 讨论

小细胞肺癌作为恶性程度更高的肺癌，手术后复发率很高且化疗效果会受限于靶向性弱、化疗耐药性等因素，致使患者预后不良，因此需要积极探究肺癌发生发展机制来寻找新型治疗靶点^[12⇓-14]^。环状RNA是人类癌症发生和恶性进展的关键调节因子，CircCCND1作为可增强CCND1基因稳定性的促癌因子^[4]^，与肝细胞癌患者相对较大的肿瘤大小和淋巴结转移有关，敲低CircCCND1可显著抑制肝细胞癌细胞的增殖、迁移和侵袭^[15]^。CircCCND1在喉癌中高表达并在其发病机制中发挥着关键作用^[16]^。Geng等^[5]^研究显示CircCCND1在肺癌细胞和患者体内显著上调，对其进行敲除可导致顺铂处理下的非小细胞肺癌细胞增殖减弱及凋亡增强，由此可知CircCCND1是治疗肺癌的潜在靶点。本研究结果显示，人肺癌H446细胞CircCCND1表达相比人正常肺上皮细胞BEAS-2B细胞明显升高，且以CircCCND1 siRNA敲低H446细胞CircCCND1表达可降低其增殖率、线粒体膜电位、迁移比、侵袭数、N-cadherin蛋白表达，同时升高其凋亡率、Bax及cleaved Caspase-3、E-cadherin蛋白表达，表明CircCCND1表达升高可能促进肺癌进展，敲低CircCCND1表达可降低肺癌细胞恶性生物学行为。

miR-340-5p作为一种具有抗癌活性的因子，在恶性肿瘤中异常低表达是促使其恶性进展的重要因素，miR-340-5p的下调可加速乳腺癌细胞周期进展，并引发其增殖行为的增强与凋亡过程的抑制^[17]^。抗癌活性物质黄芩苷可通过促进miR-340-5p的表达而对肺癌发挥肿瘤抑制作用^[18]^。TGIF1作为一种转录辅助因子，可通过调控转化生长因子β信号参与介导癌症的发生发展，过表达TGIF1可抑制食管鳞状细胞癌细胞的侵袭性表型^[19]^，TGIF1的高表达与肺腺癌患者肿瘤组织的高增殖活性和不良预后密切相关，且对于肺腺癌细胞的生存、侵袭和转移至关重要，以小分子抑制剂干扰TGIF1转录可在体外对肺腺癌细胞表现出显著的抗癌效果^[20]^，由此可知miR-340-5p和TGIF1是很有研发潜力的肺癌治疗靶点。本研究结果显示，人肺癌H446细胞miR-340-5p表达相比人正常肺上皮细胞BEAS-2B细胞降低而TGIF1表达升高，且双荧光素酶实验结果显示H446细胞中CircCCND1可靶向下调miR-340-5p表达，且miR-340-5p可靶向下调TGIF1表达，表明CircCCND1可能通过调节miR-340-5p/TGIF1轴参与肺癌的致病过程；以CircCCND1 siRNA敲低H446细胞CircCCND1表达可升高miR-340-5p表达并降低TGIF1表达，表明miR-340-5p/TGIF1轴参与敲低CircCCND1对肺癌恶性生物学行为的减弱过程；以miR-340-5p mimics上调H446细胞miR-340-5p表达可对H446细胞起到与敲低CircCCND1表达同样的抗癌作用，且CircCCND1 siRNA和miR-340-5p inhibitor联合转染可减弱CircCCND1 siRNA单独转染对H446细胞增殖、迁移及侵袭活力及线粒体膜电位的降低作用，削弱其对肺癌细胞凋亡的诱导作用，进而逆转其对肺癌细胞恶性生物学行为的抑制作用，揭示敲低CircCCND1减弱肺癌恶性生物学行为可能是通过上调miR-340-5p实现的。

综上所述，敲低CircCCND1可抑制肺癌细胞恶性行为，其作用可能是通过调控miR-340-5p/TGIF1轴实现的。本研究为肺癌的新型临床治疗靶点的开发提供了较强的理论依据，为其临床诊疗技术的改进提供了新的思路，可对肺癌患者预后的提升起到一定积极作用。未来课题组将对TGIF1进行干预，并采用其他肺癌细胞系结合体内实验进一步验证CircCCND1在肺癌中的作用与机制。
